# Multi-Omics Analysis Reveals the Pathogenesis of Growth-Disordered Raccoon Dog

**DOI:** 10.3390/ijms241814237

**Published:** 2023-09-18

**Authors:** Danyang Chen, Xiaolan Guo, Kaiying Wang, Weigang Zhao, Zhongjuan Chang, Quankai Wang, Chao Xu

**Affiliations:** 1 Institute of Special Animal and Plant Sciences, Chinese Academy of Agricultural Sciences, 4899 Juye Street, Changchun 130112, China; cdyjlau@163.com (D.C.); guoxiaolan0511@126.com (X.G.); tcswky@126.com (K.W.); zwg1163@126.com (W.Z.); changzhongjuan@caas.cn (Z.C.); 2 Innovation Center for Feeding and Utilization of Special Animals in Jilin Province and Research Center for Microbial Feed Engineering of Special Animals in Jilin Province, 4899 Juye Street, Changchun 130112, China; 3 College of Animal Science and Technology, Jilin Agriculture University, Changchun 130118, China; quankaiwang1@163.com

**Keywords:** raccoon dog, *Eperythrozoon*, transcriptome, proteome, immune

## Abstract

Microorganisms of the genus *Eperythrozoon* are a zoonotic chronic infectious disease with wide distribution. We found that raccoons infected with *Eperythrozoon* showed obvious stunting, which seriously affected the economic benefits of raccoon dogs. To investigate the pathogenesis of the raccoon dog, we used transcriptome and proteome sequencing to analyze the changes in mRNA, miRNA, and protein expression in raccoon dogs infected with *Eperythrozoon* and normal raccoons. The results showed that the expression levels of genes related to immunity, metabolism, and enzyme activity were significantly changed. Among these, *ERLIN1, IGF1R, CREB3L1, TNS1, TENC1,* and *mTOR* play key roles. Additionally, the miR-1268, miR-125b, miR-10-5p, and miR-10 as central miRNAs regulate the expression of these genes. Integrated transcriptomic and proteomic analyses revealed consistent trends in mRNA and protein changes in MYH9, FKBP1A, PRKCA, and CYP11B2. These results suggest that *Eperythrozoon* may contribute to the slow development of raccoons by affecting the expression of mRNAs and miRNAs, reducing their immunity and causing metabolic abnormalities.

## 1. Introduction

The raccoon dog (*Nyctereutes procyonoides*) is a canid that is a promising animal model [1,2]. Raccoon dogs are thought to host many pathogens [3,4,5], but remain healthy [6]. Lan et al. found that raccoon dogs may have a diversified immune system, and that most of the positively selected genes of raccoons during evolution were related to immunity, and the positive selection of these genes enabled raccoon dogs to have a diversified immune system [7]. In addition, raccoon dogs are the only canids that hibernate during the winter [8], and the adaptive evolution of their immunity may play a major role in protecting them from pathogens during hibernation [7].

Raccoon dogs are omnivorous animals [9]. They are small and fat, with disproportionately short legs and a fox-like appearance. Because of these congenital defects, raccoon dogs have learned to climb trees and swim and become omnivorous in their diet [10]. They feed mainly on various small animals, as well as plant roots, leaves, and berries from trees, fungi, and grains [11]. Some of these plants contain toxic secondary metabolites and toxic by-products from rotting food, but they can still remain healthy. However, in humans and other animals, these poisonous plants can cause gastrointestinal reactions and neurological disorders in mild cases, and death by poisoning in severe cases [12].

In China, raccoon dogs are widely bred as one of the most important fur-bearing economic animals [3]. The disease is an important factor affecting the raccoon dog industry. Although raccoon dogs carry a variety of viruses, they remain healthy, but they still have pathological conditions due to a variety of factors. There are a number of causes for the disease in animals, and studies have shown that weight loss, growth retardation, and immune deficiency in other animals have been attributed to infection with *Eperythrozoon*.

*Eperythrozoon* are pathogens that parasitize the surface of human and animal erythrocytes, plasma and bone marrow [13]. They are generally observed in smear specimens and are polymorphic in form, such as spherical, ring-, disk-, dumbbell-, racket- and comma-shaped, with fluctuating sizes, and infection with *Eperythrozoon* can have a significant impact on the health of any animal [14]. For example, Natalie J. Urie et al. found anemia and weight loss in sheep infected with *Eperythrozoon* [15]. J A Smith et al. also found transient fever, rough coat, decreased milk production, and subsequent weight loss in cows infected with *Eperythrozoon* [16]. *Eperythrozoon* is a cause of hemolytic anemia and growth retardation in animals that occurs worldwide and is currently thought to be a rickettsia [17]. In addition, some studies have demonstrated that infection by some pathogens can affect the expression levels of miRNAs and related genes, further influencing biological processes. Zhao et al. revealed that gga-miR-99a plays a key role in *Mycoplasma gallisepticum* infection through the regulation of *SMARCA5* expression [18]. 

In this study, we found raccoon dogs infected with *Eperythrozoon* and developmentally stunted in the farm. In order to investigate the pathogenesis of this disease, transcriptome sequencing, miRNA sequencing, and proteome sequencing were used to detect changes in gene and protein expression in the spleen and liver. At present, there are few studies on Eperythrozoon infection in raccoon dogs, and our experiment provides a direct basis for investigating the pathogenesis of raccoon growth disorder.

## 2. Results

### 2.1. Clinical Symptoms of Raccoons with Growth Disorders

**Figure 1:**
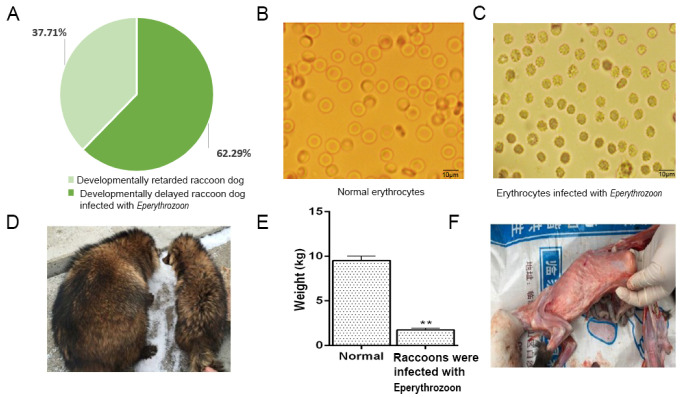
Clinical symptoms of raccoons with growth disorders. (**A**) Percentage of raccoons infected with *Eperythrozoon*. (**B**) Normal erythrocytes. (**C**) Erythrocytes infected with *Eperythrozoon*. (**D**) The weight and body size of the diseased raccoon dog are much smaller than those of the normal raccoon dog. The picture on the right shows a diseased raccoon dog. On the left is a normal raccoon dog. (**E**) Weight of a sick raccoon compared to a normal raccoon dog. Two asterisks denote significance (*p* < 0.01). (**F**) The sick raccoon dog has less subcutaneous fat.

In this study, we investigated the number of raccoons with growth retardation in the farms, and the criterion of growth retardation was that the weight of raccoons at the breeding stage was less than 50% of the average weight of the same group in the same period of time with normal or near-normal intake under normal feeding conditions. It was found that a total of 1709 raccoons out of 36,281 raccoons, or 4.71% of the total number of raccoons, had growth retardation. In order to investigate the cause of this growth disorder in raccoons, we examined 305 raccoons for infection with pathogens through testing kits and PCR. The results found that coronavirus, canine distemper virus, and microvirus were not detected in these raccoons, but the presence of *Eperythrozoon* was detected, and further analysis revealed that 62.29% of the raccoons were infected with *Eperythrozoon* (Figure 1A). *Eperythrozoon* can attach to the surface of an animal’s erythrocytes, causing the animal to develop the disease. Normal erythrocytes presented as smooth and rounded as shown in Figure 1B, while erythrocytes infected with *Eperythrozoon* appeared to be irregularly shaped and trembled as shown in Figure 1C. As shown in the figure, the stunted raccoon was much smaller in weight and size than a normal raccoon and had rough and dull fur (Figure 1D). The weight measurement showed that the normal raccoon weighed 9.75 ± 0.65 kg and the diseased raccoon weighed 1.72 ± 0.20 kg, which was significantly lower than the normal raccoon (Figure 1E). Necropsy showed that diseased raccoons were very emaciated and had very little fat under the skin (Figure 1F). These results indicate that most of the developmentally retarded raccoons were infected with *Eperythrozoon*, which suggests that *Eperythrozoon* may play an important role in pathogenicity. Therefore, the next experiments will be carried out to examine the raccoons that are stunted and infected with *Eperythrozoon* and the normal raccoons.

### 2.2. Blood Test Analysis

Next, we collected blood samples from raccoon dogs infected with *Eperythrozoon* and normal raccoon dogs for testing and analysis. The results showed that compared to the normal raccoon dogs, the number of intermediate granulocytes and Gran% were significantly higher (*p* < 0.05) and the erythrocyte pressure volume HCT was significantly lower (*p* < 0.05) in the diseased raccoon dogs (Table 1). The results of the serum samples showed that the expression levels of glutamic-pyruvic transaminase, glutamic oxalacetic transaminase, Total protein, albumin, globulin, cholesterol, high-density lipoprotein cholesterol, low-density lipoprotein cholesterol, creatine kinase, lactate dehydrogenase, serum potassium were significantly increased, while the expression levels of alkaline phosphatase, triglyceride, blood sugar, and carbon dioxide-binding power were significantly decreased, and the expression levels of total bilirubin, direct bilirubin, and indirect bilirubin were detected in the diseased raccoons (Table 2). These results show that the diseased raccoon dogs are small, poorly fatted and severely mal-nourished, while the immune function in the diseased raccoon dogs is reduced and the liver function is significantly affected. 

### 2.3. Expression Analysis and Functional Prediction of Differential mRNAs in Spleen

**Figure 2:**
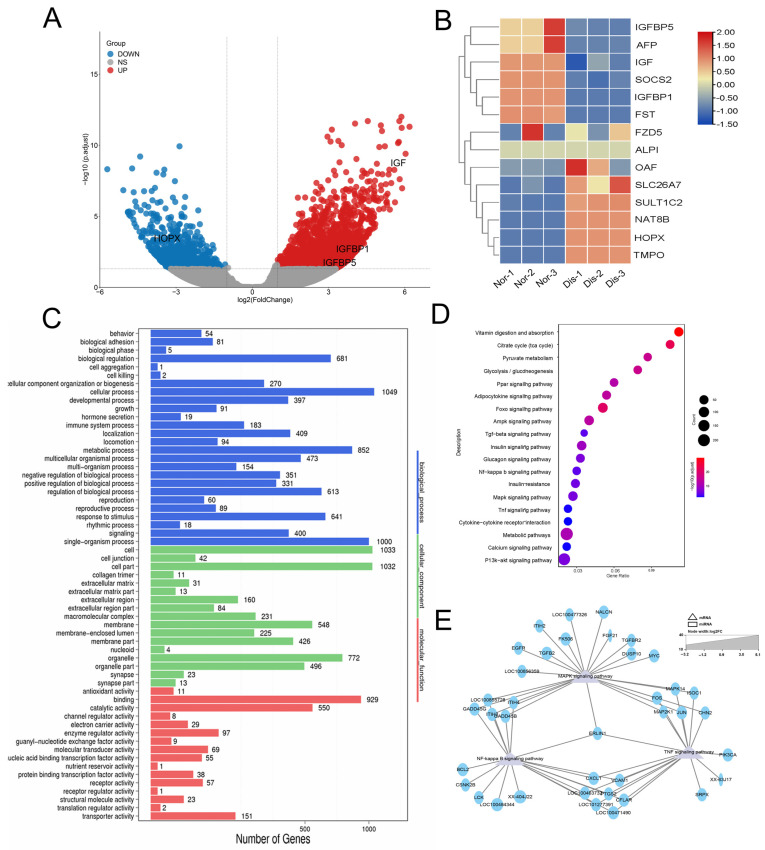
Expression analysis and functional prediction of differential mRNAs in the spleen. (**A**) Volcano diagrams of mRNA. The abscissa and ordinate represent X = log2 (fold changes) and Y = −log10 (*p*-value); the red dots indicate upregulated differential genes, the blue dots indicate downregulated differential genes, and the gray dots indicate genes with no significant difference. (**B**) The clustering heatmaps of the highest differentially expressed. (**C**) GO analysis on the function of differential genes. Nor: Normal raccoon dog, Dis: Diseased raccoon dog. (**D**) KEGG analysis of the top 20 signaling pathways enriched with differential genes. (**E**) Construction of MAPK signaling pathway, NF-κB signaling pathway, and TNF signaling pathway and related genes interactions network to find the key genes, and the results revealed that *ERLIN1* was found to be involved in all three signaling pathways simultaneously.

Transcriptome sequencing and bioinformatics analysis were conducted on the spleens of raccoons from the two groups and mRNA differential expression analysis was performed using DESeq1.28.0 software. Compared to the normal raccoon dog, there are a total of 2718 mRNA changes in the diseased raccoon dog, among which 1842 genes were up-regulated (fold-change ≥ 2) and 876 genes were down-regulated (fold-change ≤ 0.05) (Figure 2A). We screened the genes with the most significant differences for the clustering heatmap. The results showed that up-regulated genes in the spleen were mainly related to the IGF family of genes, including *IGF, IGFBP1, IGFBP5, SOCS2, FST,* and *AFP*. The down-regulated genes mainly include many enzymes such as acetyltransferase NAT8B, alkaline phosphatase ALPI, and gene expression-related genes *HOPX* and *TMPO* (Figure 2B).

To further explore the roles played by these different genes, the differential expression genes were analyzed via GO enrichment and KEGG pathways. The enrichment of target genes in the GO project includes growth, immune system process, catalytic activity, and catalytic activity (Figure 2C). Then, we performed KEGG enrichment analysis; we found that these differential genes were mainly concentrated in the MAPK signaling pathway, NF-κB signaling pathway, and TNF signaling pathway (Figure 2D). Those signaling pathways are related to immunity and development. Next, the regulatory network map was constructed, which revealed three signaling pathways to find the key genes. We found that ERLIN1 can participate in three signaling pathways simultaneously, suggesting that *ERLIN1* plays an important role in the growth and development of raccoon dogs (Figure 2E). These results showed that infection with *Eperythrozoon* significantly affected the expression of genes related to growth and development.

### 2.4. Expression Analysis and Functional Prediction of Differential mRNAs in the Liver

**Figure 3:**
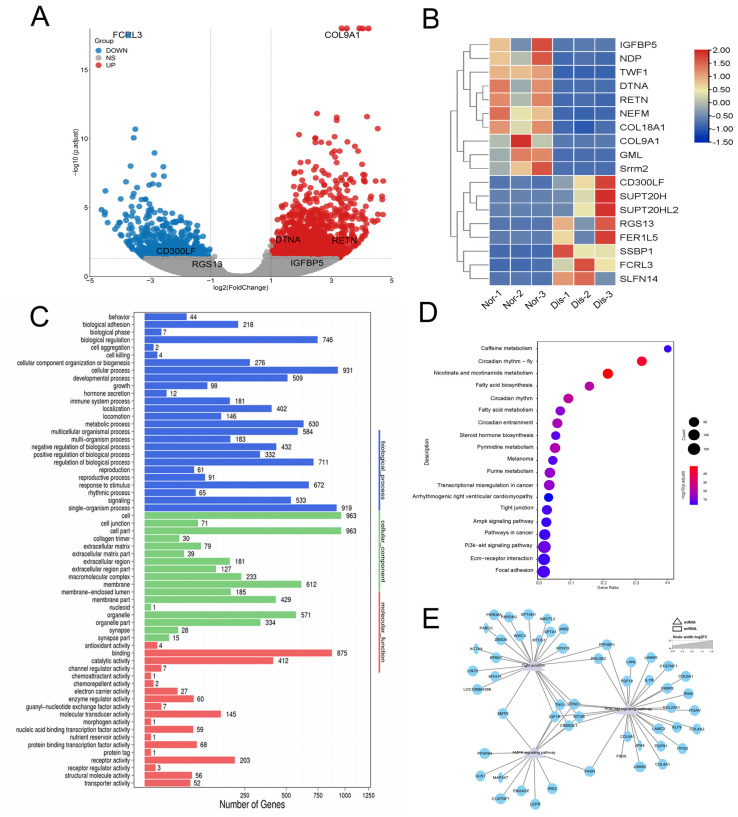
Expression analysis and functional prediction of differential mRNAs in the liver. (**A**) Volcano diagrams of mRNA. The abscissa and ordinate represent X = log2 (fold changes) and Y = −log10 (*p*-value), the red dots indicate upregulated differential genes, the blue dots indicate downregulated differential genes, and the gray dots represent genes with no significant difference. (**B**) The clustering heatmaps of the highest differentially expressed genes. Nor: Normal raccoon dog, Dis: Diseased raccoon dog. (**C**) GO analysis on the function of differential genes. (**D**) KEGG analysis of the top 20 signaling pathways enriched with differential genes. (**E**) Construction of an interplay network of PI3K-Akt signaling pathway, Tight junction, AMPK signaling pathway, and related genes aimed at obtaining key genes, and the results revealed that *IGF1R, CREB3L1, TNS1, TENC1,* and *mTOR* could participate in the three signaling pathways simultaneously.

Next, we performed a transcriptome analysis of the liver. The results showed that a total of 1996 genes were significantly changed, among which 1392 genes were up-regulated (fold-change ≥ 2) and 604 genes were down-regulated (fold-change ≤ 0.05) (Figure 3A). Similarly, we screened the genes with the largest fold change for heat mapping. The up-regulated genes mainly included *IGFBP5, RETN, DTNA, COL9A1*, etc., while the down-regulated genes mainly included immune-related genes *CD300LF, RGS13, FCRL3* (Figure 3B).

Through GO and KEGG enrichment analysis of differential genes in the liver, we found that The enrichment of target genes in the GO project includes catalytic activity, metabolic process, immune system process, and so on (Figure 3C). KEGG results showed that differential genes were mainly concentrated in PI3K-Akt signaling pathway, Tight junction, AMPK signaling pathway and some metabolic pathways (Figure 3D). Then, a regulatory network map was constructed, revealing three signaling pathways. The results revealed that *IGF1R, CREB3L1, TNS1, TENC1,* and *mTOR* were involved in the three signaling pathways (Figure 3E). These results show that infection with *Eperythrozoon* significantly affects metabolic and immune functions in the liver.

### 2.5. Analysis and Function Prediction of Differential miRNA

**Figure 4:**
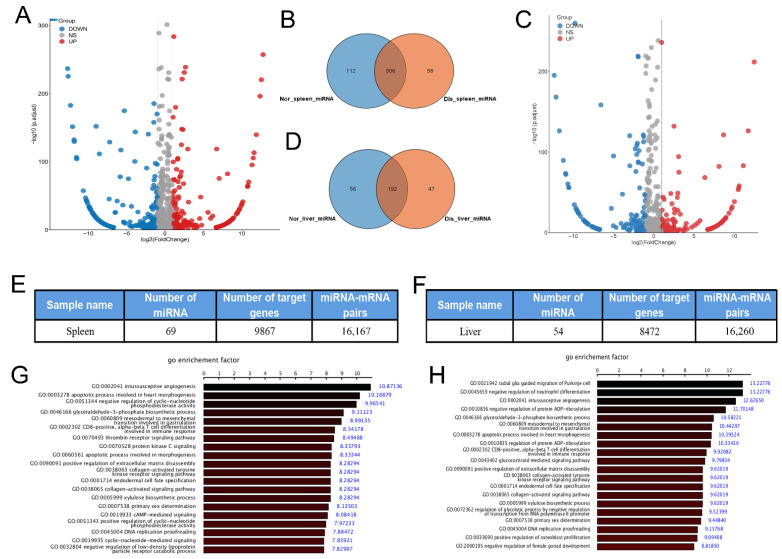
Expression analysis and function prediction of differential miRNAs. (**A**) Volcano diagrams of miRNA in the spleen; the red dots indicate up-regulated genes, the blue dots indicate down-regulated genes, and the gray dots indicate genes with no significant difference. (**B**) The distribution of differential miRNAs in the spleens of diseased and normal raccoons. The results showed that 306 miRNAs were common to both raccoons, while 58 miRNAs were specific for the diseased raccoon and 112 miRNAs were specific for the normal raccoon. Nor: Normal raccoon dog, Dis: Diseased raccoon dog. (**C**) Volcano diagrams of miRNA in the liver. The red dots indicate up-regulated genes, the blue dots indicate down-regulated genes, and the gray dots indicate genes with no significant difference. (**D**) The distribution of differential miRNAs in the spleens. The results showed that 192 miRNAs were common to both raccoons, while 47 miRNAs were specific for the diseased raccoon and 56 miRNAs were specific for the normal raccoon. (**E**) Results of miRNA and target gene count in the spleen. The results found 16,167 miRNA–mRNA pairs. (**F**) Results of the number of miRNAs and target genes in the liver. The results found 16,206 miRNA–mRNA pairs (**G**) GO analysis of the function of target genes of miRNAs in the spleen. (**H**) GO analysis of the function of target genes of miRNAs in the liver.

After clarifying the function of mRNA, we continued to analyze the expression of miRNA in the liver and spleen. The results showed that 476 miRNAs in the spleen had significant changes, with 281 miRNAs up-regulated and 195 miRNAs down-regulated (Figure 4A). Further analysis revealed that 306 miRNAs were common to both raccoon dogs, while 58 miRNAs were specific to the diseased raccoon dogs and 112 miRNAs were specific to the normal raccoon dogs (Figure 4B). A total of 294 miRNAs were found to be changed in diseased raccoons compared to normal raccoons, among which 129 miRNAs expressions were up-regulated and 165 miRNAs expressions were down-regulated (Figure 4C). Further analysis revealed that 192 miRNAs were common to both raccoons, while 47 miRNAs were specific for the diseased raccoon dogs and 56 miRNAs were specific for the normal raccoon dogs (Figure 4D).

To predict the function of these differential miRNAs, we conducted miRNA–mRNA interaction pairs to predict the function of these miRNAs through their regulated genes. The regulatory network map was constructed, which revealed 16,167 pairs of miRNA–mRNA in the spleen (Figure 4E) and 16,206 (Figure 4F) pairs of miRNA–mRNA in the liver. Later, the target mRNA were analyzed by GO enrichment. The enrichment of target genes in the GO project includes immune-related functions, such as CD8-positive, α-β T cell differentiation involved in immune response, etc. (Figure 4G). In the liver, these mRNAs are mainly enriched in immune-related functions such as negative regulation neutrophil differentiation, CD8-positive, α-β T cell differentiation, etc. (Figure 4H). These results indicate that immunity-related miRNAs were significantly changed in raccoons infected with *Eperythrozoon.*

### 2.6. Analysis of Key mRNA-miRNA Interaction Networks

**Figure 5:**
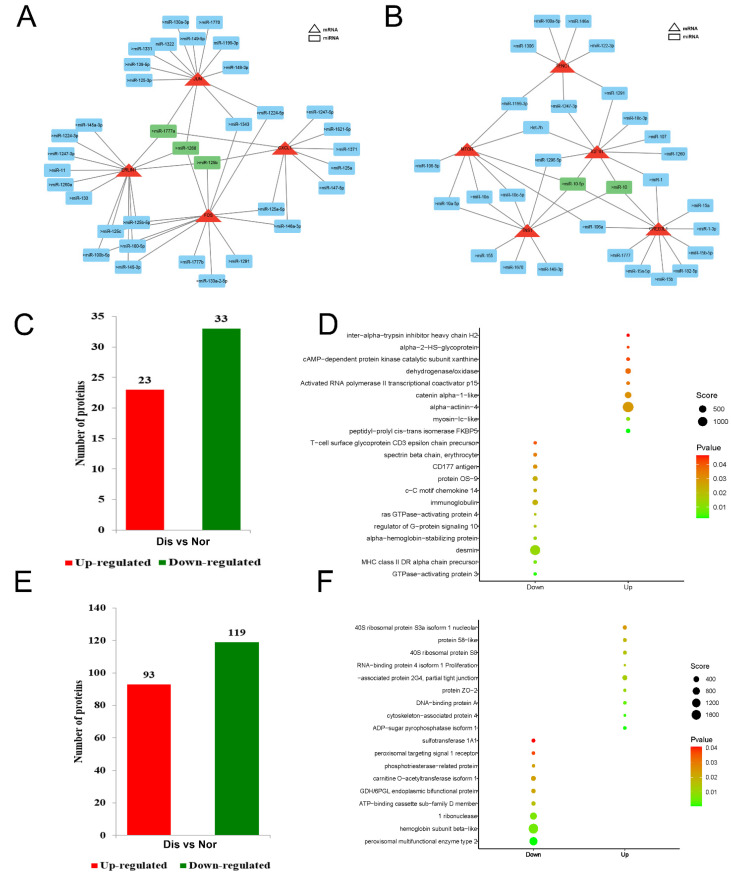
Construction of miRNA–mRNA regulatory network and differential protein analysis. (**A**) Construction of a network map of mRNA-miRNA in the spleen. The results showed miR-1268, miR-125b, and miR-1777a as central miRNAs regulating *ERLIN1, JUN,* and *FOS* genes. (**B**) Construction of a network map of mRNA-miRNA in the liver. It was found that miR-10-5p and miR-10 as central miRNAs regulate the expression of *IGF1R, CREB3L1, TNS1, mTOR* genes. (**C**) The number of differential proteins in the spleen was counted, of which 23 proteins were up-regulated and 33 proteins were down-regulated. (**D**) In the spleen, the top twenty most significantly differential proteins are shown. (**E**) A number of differential proteins are counted in the liver, of which 93 proteins are upregulated and 119 proteins are downregulated. Nor: Normal raccoon dog, Dis: Diseased raccoon dog. (**F**) In the liver, the top twenty proteins with the most significant differences are displayed.

We previously found the key genes *ERLIN1, JUN, CXCL1,* and *FOS* in the spleen. To further analyze the relationship between mRNA and miRNA, we found miRNAs that regulate these genes and the regulatory network map was constructed through the Cytoscape_3.9.1 software. We have constructed a regulatory network of key mRNA–miRNAs, and the results show that miR-1268, miR-125b, and miR-1777a act as key miRNAs to regulate *ERLIN1, JUN*, and *FOS* genes (Figure 5A). Similarly, we have analyzed the regulatory networks of key mRNA miRNAs in the liver. Then, the regulatory network map was constructed, which revealed *IGF1R, CREB3L1, TNS1, TENC1, mTOR,* and their miRNAs; the results show that miR-10-5p, and miR-10 act as central miRNAs to regulate the expression of these genes (Figure 5B). These results suggest that infection with *Eperythrozoon* resulted in significant changes in these key mRNAs, and that these mRNA changes may be closely related to the regulation of miRNAs. In summary, we found that infection with *Eperythrozoon* resulted in significant changes in mRNAs and miRNAs.

### 2.7. Differential Protein Analysis

After clarifying the changes in gene transcription levels in the diseased raccoon dog, we next performed protein-level analysis. A total of 2469 proteins were identified in the raccoon spleen via iTRAQ analysis, among which 1484 proteins could be quantified. Further analysis revealed that 56 proteins were significantly different in the spleen of diseased raccoon dogs, of which 23 proteins were up-regulated and 33 proteins were down-regulated (Figure 5C). The up-regulated proteins included activated RNA polymerase II transcriptional coactivator p15, myosin-Ib isoform 2, while the down-regulated proteins included immunoglobulin, CD177 antigen, T-cell surface glycoprotein CD3 epsilon chain precursor and other proteins (Figure 5D). 

In addition, we also analyzed the changes in protein expression in the liver. The results showed that a total of 2025 proteins were identified in the liver, among which 1282 proteins could be quantified. Further analysis revealed that the expression levels of 212 proteins were significantly changed in diseased raccoon dogs, of which 93 proteins were up-regulated and 119 proteins were down-regulated (Figure 5E). The up-regulated proteins included RNA-binding protein, proliferation-associated protein, DNA-binding protein, and down-regulated proteins included ribonuclease, hemoglobin subunit β-like and peroxidase, peroxidase and other proteins (Figure 5F). As the liver is the most important digestive and metabolic organ, we found that the expression levels of proliferation-related proteins were significantly higher in the livers of diseased raccoon dogs, and the expression levels of some enzymes were significantly lower.

### 2.8. GO and KEGG Enrichment Analysis of Differential Proteins

**Figure 6:**
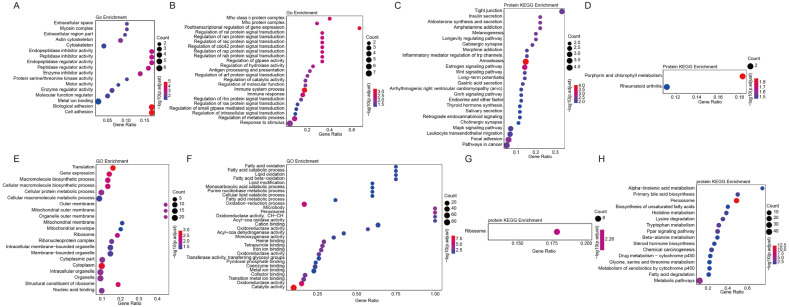
GO and KEGG analysis of the function of differential proteins. (**A**) GO analysis of the function of up-regulated proteins in the spleen. (**B**) GO analysis of the function of down-regulated proteins in the spleen. (**C**,**D**) KEGG analysis of the up-regulated and down-regulated protein enrichment signaling pathways in the spleen. (**E**) GO analysis of the function of up-regulated proteins in the liver. (**F**) GO analysis of the function of down-regulated proteins in the liver. (**G**,**H**) KEGG analysis of the signaling pathway of up-regulated and down-regulated protein enrichment in the liver.

To further analyze the functions performed by the differential proteins, we performed GO enrichment analysis on these differential proteins. The results showed that the enrichment of target genes in the GO project includes enzymatic activity and immunity, among which the up-regulated proteins were mainly concentrated in the regulation of enzymatic activity, including endopeptidase regulatory activity, enzyme regulatory activity, and cell adhesion and bioadhesion (Figure 6A), while the down-regulated proteins were mainly concentrated in the regulation of the immune system, immune response and post-transcriptional regulation and protein signaling (Figure 6B). The KEGG results showed that the differential proteins were mainly enriched in the Tight junction, MAPK signaling pathway, Wnt signaling pathway, etc. (Figure 6C,D).

In addition, we also found that the differential proteins in the liver were mainly concentrated in catalytic activity and gene expression, among which the up-regulated proteins were mainly involved in regulating gene expression and macromolecule biosynthetic process (Figure 6E), while the down-regulated proteins were mainly involved in regulating transferase activity, oxidoreductase activity and oxidation-reduction process (Figure 6F). KEGG results show that these differential proteins are mainly concentrated in the PPAR signaling pathway, metabolic pathways, peroxisome and other biological processes (Figure 6G,H). In summary, the results of GO and KEGG found that the expression levels of immune-related genes and proteins as well as signaling-related genes and proteins were significantly decreased in the spleen of diseased raccoons, while gene expression and synthesis-related functions were increased and the activity of some enzymes was decreased in the liver. 

### 2.9. Construction of Differential Gene and Protein Regulatory Networks

**Figure 7:**
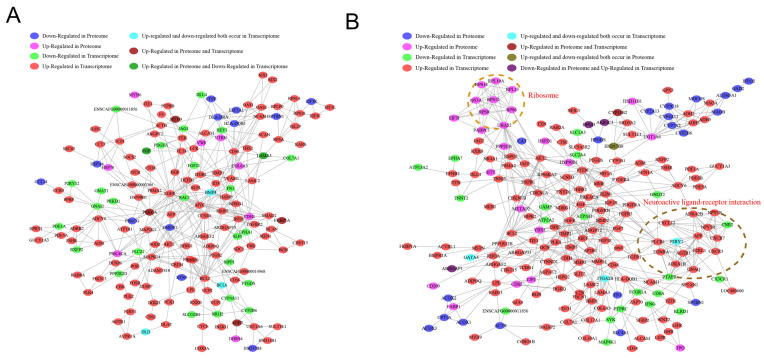
Constructing gene–protein interaction networks based on transcriptomic proteomic association analysis. (**A**) Construction of a differential gene and differential protein inter-regulatory network in the spleen. (**B**) Construction of a differential gene and differential protein regulatory network in the liver.

To better describe the correlation of gene and protein changes in the spleen and liver of raccoon dogs, we performed a co-analysis of transcriptomic and proteomic sequencing data and constructed a network of gene and protein interaction in the spleen and liver, respectively. The combined analysis identified genes that are up-regulated in both the transcriptome and proteome of the spleen, including MYH9, FKBP1A, and PRKCA (Figure 7A), which are mainly involved in immune and metabolic functions. Next, we identified the CYP11B2, which is up-regulated in both the transcriptome and proteome of the liver, (Figure 7B) with functions such as catalysis of iron binding and oxidoreductase activity. This also suggests that the activity and function of some enzymes in the liver are also significantly affected. These results again indicate that infection with *Eperythrozoon* significantly affects the function of the spleen and liver of raccoons, in which MYH9, FKBP1A, and PRKCA, and CYP11B2 are significantly changed at the protein and gene levels, and that these genes play important roles in growth and development, and immunity. These results provide a theoretical basis for understanding the pathogenesis of growth disorders in raccoons infected with *Eperythrozoon*.

## 3. Discussion

Raccoon dogs are economically valuable furbearers [19]. During the survey of the farm, we found that there were many raccoons with developmental disorders, and these raccoons had a significant loss of weight compared to normal raccoons, and further analysis revealed that 62% of the diseased raccoons were infected with *Eperythrozoon*. In order to investigate the pathogenesis of *Eperythrozoon* infection in raccoons, we first performed blood analysis and found that the number of granulocytes was significantly reduced in sick raccoons, and granulocytes have a significant role in regulating adaptive immunity [20]. In addition, we found that liver function was significantly affected in sick raccoons. Next, in this study, transcriptomic and proteomic analyses of raccoon liver and spleen tissues were performed to reveal the changes in mRNA and protein expression after infection with epizootic *Eperythrozoon*.

Transcriptome sequencing analysis reveals a large number of differential genes in the spleen of diseased raccoons, among which the expression levels of IGF family genes, including *IGF, IGFBP1* and *IGFBP5*, were significantly altered. These genes are involved in the regulation of growth and development, and the IGF family also has a role in the regulation of immunity and inflammation [21,22,23,24]. In addition, we have found significant effects on signal transduction, digestion, metabolism, cellular communities, and immune system functions in the spleen of the diseased raccoons. The differential genes in the liver transcriptome data mainly include *IGFBP5, RETN, DTNA,* and *COL9A1*. These differential genes are also primarily associated with immunity and malnutrition. *IGFBP5* has been found to promote cell growth and to be involved in immune regulation [25,26]. Dysregulation of *RETN* gene expression can lead to acquired generalized lipodystrophy disease, and *RETN* is also involved in immune regulation [27]. *DTNA* is a member of the myotonic dystrophy protein subfamily of the myotonic dystrophy protein family. Dysregulation of *COL9A1* expression may lead to the development of the disease [28]. We also found significant changes in metabolic and signal-related functions in the liver of diseased raccoons. These results indicate that immune regulation in the spleen is significantly reduced, which may be associated with changes in gene expression, while the liver is mainly associated with reduced metabolic and signal-related functions. In conclusion, these results indicate that raccoons infected with *Eperythrozoon* have significantly reduced immunocompetence as well as metabolic abnormalities.

Through the interaction network map to exploit more key genes, we found that *ERLIN1* can regulate the PI3K-Akt signaling pathway, NF-κ B signaling pathway, and TNF signaling pathway in the spleen, while it has been shown that *ERLIN1* plays an important role in development [29] and that *ERLIN1* has been identified as a significant connecting bridge between immunity and metabolism [30]. miRNAs, as small non-coding RNAs, can regulate the expression of mRNAs involved in biological processes. In this study, we analyzed the regulatory role of miRNAs with mRNAs and revealed that miR-1268, miR-125b, and miR-1777a can regulate *ERLIN1, Fos*, and *Jun* genes, which are all related to growth and development. In addition, *IGF1R, TNS1,* and *mTOR* were found to regulate the PI3K-Akt signaling pathway, Tight junction, and AMPK signaling pathway in the liver, among which *IGF1R* is a well-known regulator of cell growth, differentiation, metabolism, and function [31]; *TNS1* is involved in various biological processes such as cell adhesion, polarization, migration, invasion, proliferation, and apoptosis by interacting with various associate proteins [32]. The aberrant expression of *TNS1* can cause a variety of diseases by affecting downstream signaling pathways such as Rho GTP and PI3K/Akt/mTOR signaling pathways [33]. While miR-10-5p and miR-10 regulated the expression of these genes, which may indicate that these miRNAs can regulate the expression of genes and thus affect signaling. Furthermore, we found that miRNAs are involved in the regulation of immune-related genes, both in the spleen and in the liver, and that these genes are mainly enriched in immune-related functions such as CD8-positive, α-βT cell differentiation involved in the immune response. In conclusion, these results indicate that immunity-, metabolism-, and growth-associated mRNAs and miRNAs were significantly changed in raccoons infected with *Eperythrozoon*, suggesting that the pathogens may regulate the immune system, metabolism and growth of the organism by affecting the expression of mRNAs and miRNAs.

Finally, combined transcriptomic and proteomic analyses showed that MYH9, FKBP1A, PRKCA and, CYP11B2 were significantly changed in both the transcriptome and proteome, and that these genes and proteins were all associated with development and immunity. MYH9 genes play a role in the cytoskeleton, cell polarity, and cell motility, and are involved in protein secretion and vascular endothelial cell migration [34,35,36]. Meanwhile, in humans, MYH9 also plays an important role, as MYH9 mutation leads to thrombocytopenia. This type of patient presents chronic or intermittent elevation of liver enzymes, especially transaminases [37]. In our study, we also found that the ability of the liver to synthesize enzymes was affected in raccoons infected with *Eperythrozoon*. KBP1A functions as an immunosuppressant in combination with FK506 and inhibits calcium-modulated neurophosphatase activity via FKBP/FK506 [38,39,40]. In addition, it has been shown that FKBP1A has an important role in tumor development. The FKBP1A/Rapamycin complex acts on the mammalian target of Rapamycin (mTOR) by repressing its serine/threonine phosphatase activity to achieve the effect of cell cycle arrest and cell growth repression [41]. The cell cycle is closely related to the growth and development of the organism, which also suggests that the KBP1A gene regulates growth and development in both humans or raccoons dogs. PRKCA belongs to the protein kinase C (PKC) family, which is involved in regulation of cell proliferation, apoptosis, differentiation, migration, cardiac hypertrophy, and inflammation. PRKCA is expressed in all human tissues, and it has been shown that PRKCA is associated with cardiovascular disease, body weight, and bone structure [42]. In our experiments, we also found abnormal PRKCA gene expression in raccoons with developmental delay, which suggests that the PRKCA gene has a regulatory role in the health of both humans and animals. In addition, the CYP11B2 gene was also significantly altered. The function of CYP11B2 gene is closely related to growth and development, and some studies have shown that patients deficient in CYP11B2 from 4 to 11 months of age suffer from vomiting, slow weight gain, hyponatremia, hyperkalemia, and low aldosterone levels [43]. We also found significant changes in the expression of this gene in raccoons with developmental delays. In summary, we found that these genes can affect the health of raccoons and humans, and maintaining the stability of these genes is essential for growth and development.

Based on these studies, we found that infection with *Eperythrozoon* significantly affected development, immune-related genes and miRNA changes, in which the IGF family may play an important role. At the protein level, the expression levels of immune proteins, metabolic proteins and transcriptional regulation-related proteins were significantly altered in the spleen. Additionally, the major proteins that were changed in the liver were associated with enzyme activities. In addition, by combining the transcriptomic and proteomic analyses, we found consistent trends of mRNA and protein changes in the MYH9, FKBP1A, PRKCA, and CYP11B2, which are all associated with the functions of immunity, metabolism, and enzyme activity. These results suggest that *Eperythrozoon* may cause weight loss and growth disorders in raccoons by decreasing their immunity and causing abnormalities in their metabolism.

In summary, the results of this study provide some reference data for elucidating the gene and protein expression changes in raccoon after infection with *Eperythrozoon*, and for exploring the pathogenic mechanisms of raccoon growth disorders. In the future, we will also keep exploring the causes of other developmental retardation raccoons to further supplement all the causes of developmental retardation as well as to clarify other etiologies of developmentally delayed raccoons and their gene and protein changes.

## 4. Materials and Methods

### 4.1. Sample Collection

On farms, during the rapid growth period of raccoons, a raccoon with a normal or near-normal intake of less than 50% of the average weight of the same group is called a stunted raccoon. The clinical characteristics of a sick raccoon are lethargy, small size, anemia, loose hair, slow growth, and weight loss. In this study, normal raccoons and sick raccoons were selected at 7 months of age, at the same growth period, and their weights were measured separately. Afterwards, we used microscopic venous blood smear examination for *Eperythrozoon* detection (at least one *Eperythrozoon* per 20 vision fields of the microscope or per 200 erythrocytes) [14].

Three liver tissues and spleen tissues were collected from normal and diseased raccoons dogs during the same period. The obtained tissues were cut into small pieces and divided into centrifugal tubes for rapid freezing. The rapidly frozen tissue blocks were then used for RNA-seq, miRNA, and proteomic analysis. The research was approved by the Institute of Special Animal and Plant Sciences of the Chinese Academy of Agricultural Sciences (license number ISAPSAEC-2021-61RD).

### 4.2. Blood Test

Blood samples were collected from raccoon dogs in each group using 10 mL EDTA (Procell Life Science&Technology Co., Ltd., Wuhan, China) anticoagulant tubes. After collecting the blood, the blood collection tube was slightly inverted and placed vertically in a centrifuge tube rack. The EDTA anticoagulated tubes containing blood were placed in a centrifuge at 3500 rpm, 4 °C for 10 min, and the supernatant was aspirated to obtain the raccoon dogs’ serum and plasma, which was subsequently dispensed into centrifuge tubes. One blood sample was used for routine blood tests and one for physiological and biochemical parameters. Finally, blood cells, liver function, lipids, electrolytes, bilirubin and glutamic-pyruvic transaminase, glutamic oxalacetic transaminase, total protein, albumin, globulin, cholesterol, high-density lipoprotein cholesterol, low-density lipoprotein cholesterol, creatine kinase, lactate dehydrogenase, serum potassium, alkaline phosphatase, triglyceride, blood sugar, and carbon dioxide binding power, total bilirubin, direct bilirubin, and indirect bilirubin were obtained using the Beckman AU480 automatic biochemistry analyzer (Vitalab Selectra E, Spankeren, The Netherlands).

### 4.3. Library Construction and RNA Sequencing

Total RNA was extracted from the liver and spleen tissues of raccoon dogs using an RNA extraction kit (Takara Bio Inc., Dalian, China). The extracted RNA was enriched with magnetic beads with Oligo (DT) from raccoon liver and spleen; then, the enriched mRNA was broken into shorter fragments, and the cDNA was synthesized using the obtained mRNA fragments as templates with random primers, and finally purified using the QiaQuick PCR kit (Qiagen, Germany), followed by sequencing using the Illumina Hiseq 2000 platform. The data were filtered based on raw reading output from the Illumina Hiseq 2000 platform. All downstream analyses were based on high-quality clean reads. The filtered clean reads were then spliced and compared with the database using Trinity3 software. The samples from each group were then analyzed for differential expression using DESeq1.28.0 [44].

### 4.4. miRNA Sequencing and Analysis

miRNA sequencing was performed in the spleen and liver of normal raccoon dogs and diseased raccoon dogs. Firstly, raw data were obtained based on HiSeq high-throughput sequencing technology, and the reads with lengths less than 18 nt were further filtered and removed; finally, the clean reads were obtained after de-functioning and decontamination. Next, the clean reads were compared with the genome and annotated for classification to clarify the miRNA classes.

Bioinformatics was applied to analyze the differentially expressed miRNAs in different tissues. The criteria of screening for the differences in the miRNA expression were *p*-value ≤ 0.05. Next, the number of miRNAs common and unique to the two tissues was analyzed using Tbtools 1.112 software. Target gene prediction software Miranda (3.3a) was used to confirm the target genes of these different miRNAs. After that, GO and KEGG functional annotation was carried out on the target genes. Finally, Cytoscape 3.6.1 software was used to construct key miRNA–mRNA interaction pairs to analyze the roles of miRNA–mRNA in raccoon dogs.

### 4.5. Proteomic Analysis

The liver and spleen tissues were ground into powder using liquid nitrogen, and then the sample powder was transferred to a 5 mL centrifuge tube. Then, 8 mol/L urea, 2 mol/L EDTA, 10 mol/L DTT, and 1% protease inhibitor were added into the centrifuge tube. After centrifugation, the supernatant was obtained and the protein concentration was measured. Finally, trypsin digestion was performed. After the trypsin-digested peptide was dehydrated and vacuum-dried, the peptide was redissolved in 0.5 mol/L TEAB and protein labeling was performed according to the instructions of the 8-plexi TRAQ kit (sigma-aldrich, Saint Louis, MO, USA). Briefly, one unit of iTRAQ reagent (defined as the amount of reagent required to label 100 μg of protein) was thawed and reconstituted in 24 μL ACN. The peptide mixtures were then incubated for 2 h at room temperature and pooled, desalted, and dried by vacuum centrifugation. The sample was then fractionated into fractions via high pH reverse-phase HPLC using Agilent 300 Extend C18 column (5 μm particles, 4.6 mm ID, 250 mm length). Finally, and dried via vacuum-centrifuging.

### 4.6. LC-MS/MS Proteome Quantitative Analysis

Peptides were dissolved in 0.1% FA, and directly loaded onto a reversed-phase precolumn (Acclaim PepMap RSLC columns, Thermo Fisher Scientific, 81 Wyman Street, Waltham, MA, USA). The resulting peptides were analyzed via Q ExactiveTM plus hybrid quadrupole-Orbitrap mass spectrometer (ThermoFisher Scientific). The peptides were subjected to an NSI source followed by tandem mass spectrometry (MS/MS) in Q ExactiveTM plus (Thermo) coupled online to the UPLC. Finally, MS/MS data were obtained using Mascot (v.2.3.0) [45], and the sequenced proteins were quantified. The quantitative ratio ≥ 1.2 was regarded as up-regulated, while the ratio ≤ 0.83 was regarded as down-regulated.

### 4.7. GO and KEGG Analysis

The gene ontology (GO) annotation proteome was derived from the UniProt-GOA database. Then, proteins were classified via Gene Ontology annotation based on three categories: biological process, cellular component, and molecular. GO functional terms of differentially expressed genes, miRNAs, and proteins were enriched using KOBAS (2.0) software to analyze GO enrichment [46,47]. Kyoto Encyclopedia of Genes and Genomes pathway enrichment analysis [48,49,50] was carried out.

### 4.8. Combined Transcriptome and Proteome Analysis

Cytoscape_3.9.1 software in the STRING 10.0 database [51] was used to analyze the relationship between differential proteins and differential transcripts. The proteins for analysis were those conforming to quantitative conditions, and the standard was, when quantitative, non-repeating polypeptide ≥ 2, the fold change of differential proteins was ≥1.2, and the fold change of differential genes was ≥2.

### 4.9. Statistical Analysis

The experimental data were presented as means ± standard deviation (SD). SPSS 22.0 software (IBM, Armonk, NY, USA) was used to perform all the statistical analyses. The significant differences between the groups were evaluated using Student’s *t*-test. The *p* values < 0.05 was considered statistically significant.

## Figures and Tables

**Table 1:** The test results of regular blood (disease vs. normal).

Item	Normal	Disease
WBC (×10^−9^/L)	15.3 ± 8.91	18.06 ± 12.41
Lymph (×10^−9^/L)	0.93 ± 1.32	3.57 ± 4.97
Gran (×10^−9^/L)	-	4.567 ± 2.83 *
Gran (×10^−9^/L)	-	17.4 ± 13.86
Lymph %	0.089 ± 0.1258	27.43 ± 43.36
Mid %	-	16.27 ± 4.91 **
Gran %	-	56.3 ± 38.45 *
RBC (×10^−9^/L)	7.43 ± 0.96	5.41 ± 1.74
HGB (g/L)	130.6 ± 39.39	102 ± 34.29
HCT (L/L)	45 ± 6.09	31.93 ± 10.43 *
MCV (fL)	60.57 ± 1.087	59.13 ± 3.14
MCH (pg)	19.7 ± 0.67	18.76 ± 1.21
MCHC (g/L)	325.34 ± 4.92	318.29 ± 8.04
RDW-CV%	14.88 ± 0.29	14.94 ± 1.35
RDW-SD (fL)	33.37 ± 1.08	33.06 ± 2.58
PLT (×10^−9^/L)	525.67 ± 136.76	660.71 ± 305.30
MPV (fL)	9.7 ± 0.37	9 ± 0.68
PDW %	15.3 ± 0.51	15.76 ± 0.23
PCT %	0.51 ± 0.12	0.61 ± 0.31

Diseased raccoons compared to normal raccoons, * represents *p* < 0.05; ** represents *p* < 0.01.

**Table 2:** The test results of blood biochemical criteria (disease vs. normal).

Item	Normal	Disease
glutamic-pyruvic transaminase (IU/L)	33.0 ± 2.6	570.5 ± 22.6 **
glutamic oxalacetic transaminase (IU/L)	45 ± 4.6	161 ± 9.8 **
Total protein (g/L)	67.8 ± 5.9	116.4 ± 13.8 **
albumin (g/L)	31.7 ± 6.3	50.6 ± 7.1 **
globulin (g/L)	36.1 ± 5.5	65.8 ± 6.8 ** (g/L)
Alkaline phosphatase (IU/L)	59.1 ± 7.7	29.3 ± 6.4 * (IU/L)
triglyceride (mmol/L)	1.43 ± 0.32	0.61 ± 0.24 *
Cholesterol (mmol/L)	3.84 ± 0.55	5.29 ± 0.78 *
High density lipoprotein cholesterol (mmol/L)	3.25 ± 0.32	4.63 ± 0.56 *
Low density lipoprotein cholesterol (mmol/L)	0.07 ± 0.03	0.89 ± 0.22 **
Creatine kinase (IU/L)	293.0 ± 32.1	856.0 ± 44.2 **
Lactate dehydrogenase (IU/L)	153 ± 22	502 ± 43 **
Blood sugar (mmol/L)	4.49 ± 0.88	2.39 ± 0.69 **
Serum potassium (mmol/L)	5.72 ± 0.64	15.04 ± 1.23 **
Blood sodium (mmol/L)	143 ± 22	158 ± 18
Blood chlorine (mmol/L)	102 ± 9	117 ± 11
Carbon dioxide binding power (mmol/L)	26.3 ± 1.2	10.1 ± 1.4 **
Blood calcium (mmol/L)	2.84 ± 0.21	2.94 ± 0.32
Total bilirubin (μmol/L)	-	1.2 ± 0.1
Direct bilirubin (μmol/L)	-	1.0 ± 0.2
Indirect bilirubin (μmol/L)	-	0.2 ± 0.01

Diseased raccoons compared to normal raccoons, * represents *p* < 0.05; ** represents *p* < 0.01.

## Data Availability

The original contributions presented in the study are included in the article, and further inquiries can be directed to the corresponding author.
